# Extrusion-based additive manufacturing of forming and molding tools

**DOI:** 10.1007/s00170-021-07162-8

**Published:** 2021-05-04

**Authors:** Matteo Strano, Kedarnath Rane, Muhammad Asad Farid, Valerio Mussi, Veronica Zaragoza, Michele Monno

**Affiliations:** 1 Dipartimento di Meccanica, Politecnico di Milano, Milano, Italy; 2 MUSP – Macchine Utensili e Sistemi di Produzione, Piacenza, Italy

**Keywords:** Extrusion additive manufacturing, Rapid tools, Forming, Shearing, Bending, Drawing, Molding, Thermoplastics, Metals, Ceramics, FFF, EAM

## Abstract

The production of rapid tools for plastic molding, sheet metal forming, and blanking has always been a critical and important goal for applied research, and a very large number of alternative methods have been proposed over the decades for their production. Among these methods, the use of extrusion-based additive manufacturing (EAM), such as fused filament fabrication (FFF) or similar technologies, has not been frequently considered and needs to be explored extensively. EAM is generally considered a low-cost, low-quality, low-performance class of AM and not suited to produce real functional parts, but only for aesthetical prototypes. However, the capabilities of EAM technologies have greatly evolved and now it is possible to extrude a wide range of materials such as polymeric materials including both the low strength polymeric materials (such as nylon or PLA) and the high strength polymeric materials (such as PEI and PEEK), metals (such as tool steel), and even ceramics (such as zirconia). Starting from an extensive literature review, the purpose of the present paper is to further demonstrate the potential applicability and versatility of EAM as a rapid tool manufacturing technology for different applications in shearing, bending, deep drawing, and injection molding.

## Introduction

Nowadays, additive manufacturing (AM) methods are commonly used in many different fields such as aerospace, automotive, and medical just to name a few. Currently, the automotive and aeronautical sectors are shifting their focus to a more customized production, thus facing an increasing demand for medium- and small-batch productions, where the necessity of flexible or rapid tools takes higher importance [1]. For instance, in the automobile industry, injection molding and sheet metal forming are widely used. Conventional tooling is suitable only for mass production; in the case of batch or customized production, traditional tooling becomes cost-prohibitive highlighting the importance of rapid tooling in such scenarios to achieve cost-effectiveness and reduction in lead time. The importance of rapid tools has emerged as a dramatic method for increased resilience of industrial factories, in the case when they need to rapidly reconvert their production to new devices, such as PPE (personal protection equipment) which suffered a shortage under the COVID-19 pandemic. Rapid tools can very effectively exploit the flexibility and cost efficiency of AM technologies, for instance, the fused filament fabrication (FFF). FFF is one of the most common techniques used for 3D printers and has become one of the most popular rapid prototyping (RP) techniques in the last decade [2]. A more general and comprehensive definition of the technology is extrusion-based additive manufacturing (EAM). Among the AM methods, EAM provides an alternative production process to create parts in a fast way and at a lower cost compared to other existing methods [3].

This paper, therefore, directs the attention to the usability of EAM techniques in the rapid tooling domain. It intends to illustrate the application potential and flexibility of the EAM techniques for sheet metal operations and injection molding by taking advantage of the availability and suitability of the wide range of metallic and polymeric feedstocks with EAM.

Material extrusion technology is a layered production method where the material is selectively distributed on a nozzle or orifice side. In the material extrusion system, the raw material is melted in the extrusion head and poured selectively onto the print bed by the nozzle, or this movement can be achieved by moving the printing area table in the x-y plane. After a layer is completed, the construction platform moves downward, or the extrusion head moves and presses the new layer onto the previous layer. EAM can be used to produce rapid tools or parts using several different materials:

- thermoplastics [4];
- short fiber reinforced thermoplastics [5];
- metals [6];
- technical ceramics [7];
- cermets and hardmetals [8].

All of these kinds of materials can be used for rapid tooling aimed at low batch productions. Moreover, intensive research is being carried out to enhance the applicability of material extrusion AM by developing new materials [9].

In EAM, the material feedstock can be provided to the machine in the form of pellets or in the form of a filament, which is the traditional FFF technology [10]. On the contrary, the usage of EAM for metals and ceramics is not widespread; however, it is increasingly being employed for the rapid production of metals and ceramic components, and the published scientific and technical literature on it is surging. The EAM production route for metals and ceramics will be presented in Section 2.

Conventional tools, dies, and molds used in sheet metal forming or injection molding industries are made of metal, generally, tool steel, because their expected life should be long, and their mechanical and thermal performance must be enduring. The most important mechanical, thermal, and geometrical properties of tools are the following:

- Compression strength
- Elastic modulus
- Poisson coefficient
- Fatigue strength
- Thermal diffusivity
- Hardness at room temperature and at high temperature
- Surface roughness
- Geometrical tolerances

3D printed tools by EAM do not easily meet any of the requirement, and this poses a significant challenge for the manufacturing industry. Conventional metal tools more easily fulfill the abovementioned mechanical specifications, and also the geometrical properties, because they are machined and, in most cases, they are also ground to the desired precision.

## Classification of rapid tools

Rapidly manufactured tools (herein after called rapid tools for brevity) can be categorized into two distinct classes: soft tools and hard tools. Soft tools comprise of materials that are softer and easier to process than steel for example low-temperature alloys and aluminum; hard tools are made from tool steel. There are significant differences between the mechanical and thermal properties of soft and hard tools affecting the tool life and the final part quality. Therefore, soft tools are suitable for short manufacturing runs [11].

### Metal EAM rapid tools

Metallic rapid tools have successfully been 3D printed in the past for forming and molding applications. Extensive literature is available related to rapid metallic tools manufactured using layer-assisted additive manufacturing processes. A wide range of metals has been considered in this regard by various authors. However, the fabrication of the tools has been limited to physical prototyping and small quantity production yet [12]. This paper deals with the potential applicability and feasibility of using EAM for rapid metallic tools and provides the fundamental concepts for their manufacturing.

The EAM process has been developed in order to print metallic parts. The principle is the same as of the conventional FFF but, in this case, the feedstock is made by around 50 or 60% vol. of metallic powders that are surrounded by a continuous thermoplastic matrix [13]. The feedstock, once heated, passes through the nozzle in a viscous state and it is deposed as in standard FFF [14]. The feedstock can be in the form of a filament, or pellets, or solid rods [15].

Once deposited, the part cools down and solidifies. At this stage, the object is a mixture of polymer and metal; therefore, 1 or 2 debinding stages followed by a sintering stage are performed to obtain dense metallic parts, which shrink with respect to their dimensions at the green state [16]. This allows removing the polymeric fraction obtaining a solid component. The final sintered quality of the parts (surface roughness and porosity) is better than the quality of conventional FFF [17].

Some of the leading 3D printer manufacturers have advanced significantly in providing solutions for successful extrusion-based additive manufacturing of metals. For example, desktop metal is able to extrude metallic components using metal injection molding (MIM) as its enabling technology [18]. They claim to be able to 3D print tools made of H13 steel. Another company (Markforged) with its Metal X 3D printer [19] claims to achieve a final density of printed parts of 99.7% made by 17-4 PH stainless steel, H13 tool steel, Inconel 625, and Ti-6Al-4V.

Virtually any kind of metal can be 3D printed with this technology of EAM, including the most conventional tool steel and stainless steel.

### Polymeric EAM rapid tools for sheet metal forming

In almost all the major manufacturing industries, sheet metal operations have been employed for producing different components for several decades. Traditionally, the tools were designed and manufactured keeping in mind the ability to mass-produce a large number of one type of product which justified the tooling cost but, in a batch, or customized part production, where sheet thickness and material may change very frequently, changing the die and the punch for each specific type of product is just not economical. For this reason, the concept of rapid tools emerged not less than 30 years ago [20]. Recently, the interest in rapid and flexible tools is growing again, due to the improved quality of additive manufacturing technologies and materials. The growing demand in the reduction of production time and costs increases the need for faster response times and more efficient means to produce prototypes and tools in the short term.

As a result, the use of rapid tool technologies, such as AM, using advanced polymeric materials and composite materials to manufacture sheet forming dies arises with the aim of reducing the delivery time and the investment cost of tool development.

Successful attempts have been made to employ the polymeric rapid tools for sheet metal operations with desirable final part characteristics. Nakamura et al. analyzed the efficiency of the V-bending process for aluminum and steel sheets using different combinations of tools made of 3D printed PLA and steel; their study showed that plastic tools can be used to bend metal sheets, being noticed that to improve the dimensional accuracy of the products it is effective to use a combination with a steel punch and a plastic die [21]. Kuo and Li 3D printed a die and a punch for sheet metal forming using FFF. The material used for tooling was ABS. The tooling was tested using a hydraulic machine for forming of Al–Mg alloy sheets with a thickness of 0.6 mm. The tooling was proven to be effective experimentally and was found to be rigid enough to fulfill small batch production with good dimensional accuracies [22]. Schuh et al. analyzed the suitability of 3D printed PLA tools for sheet metal. It was observed that the PLA tools can provide similarly good results in formability as compared to the metallic tools [23].

All these efforts motivate the exploration of EAM for 3D printing of polymeric rapid tools. However, the strength of parts manufactured by EAM is lower than the ones obtained with other manufacturing techniques (for example, injection molding), which affects the bend angles obtained with respect to steel tools. For this reason, it is desirable to develop an approach to reduce the elastic deformation of plastic tools made by EAM.

In the literature, some approaches can be found with respect to improving the strength of 3D printing components. Some authors have studied the influence of printing parameters (layer thickness, orientation, raster angle, raster width, and air gap) on the mechanical properties of the tool (tensile, flexure, and impact strengths). Among these parameters, it has been found that the build orientation, the raster parameters, and the air gap are the ones that can influence more the mechanical properties of the part [24, 25]. Using ABS samples, Hernandez et al. evaluated the effect of 5 different printing orientations in a plane XYZ, finding that the samples printed at 0° in the XY plane offer the strongest resistance in compression and flexure, having the greatest modulus of elasticity, while the samples printed at 90° in the XY plane shows the strongest behavior in tension, having the largest tensile strength and lowest modulus of elasticity. The raster angle used by default in EAM is 45°–45°. However, there is an important interaction between the build mechanisms in EAM which does not allow to establish a single independent parameter as the best condition to obtain higher performances [26]. Furthermore, there cannot be any general rule because the required mechanical properties depend on how the tool will be used and what kind of stresses it will bear.

Other methods to improve the strength of EAM printing materials, independent of the printing parameters, also include the use of filler materials such as carbon fibers or composite resins as an addition to the printing process. The layers and the direction of the fibers introduce an anisotropic effect that greatly influences the strength of the 3D printed part in a given direction. Belter et al., for example, showed a technique to increase the strength in the manufacture of thermoplastic parts by EAM, carefully placing holes in the printed parts and filling them with high-strength resins. This system is limited by the strength of the thermoplastic and offers only slight improvements over standard EAM printing methods [27].

**Fig. 1:**
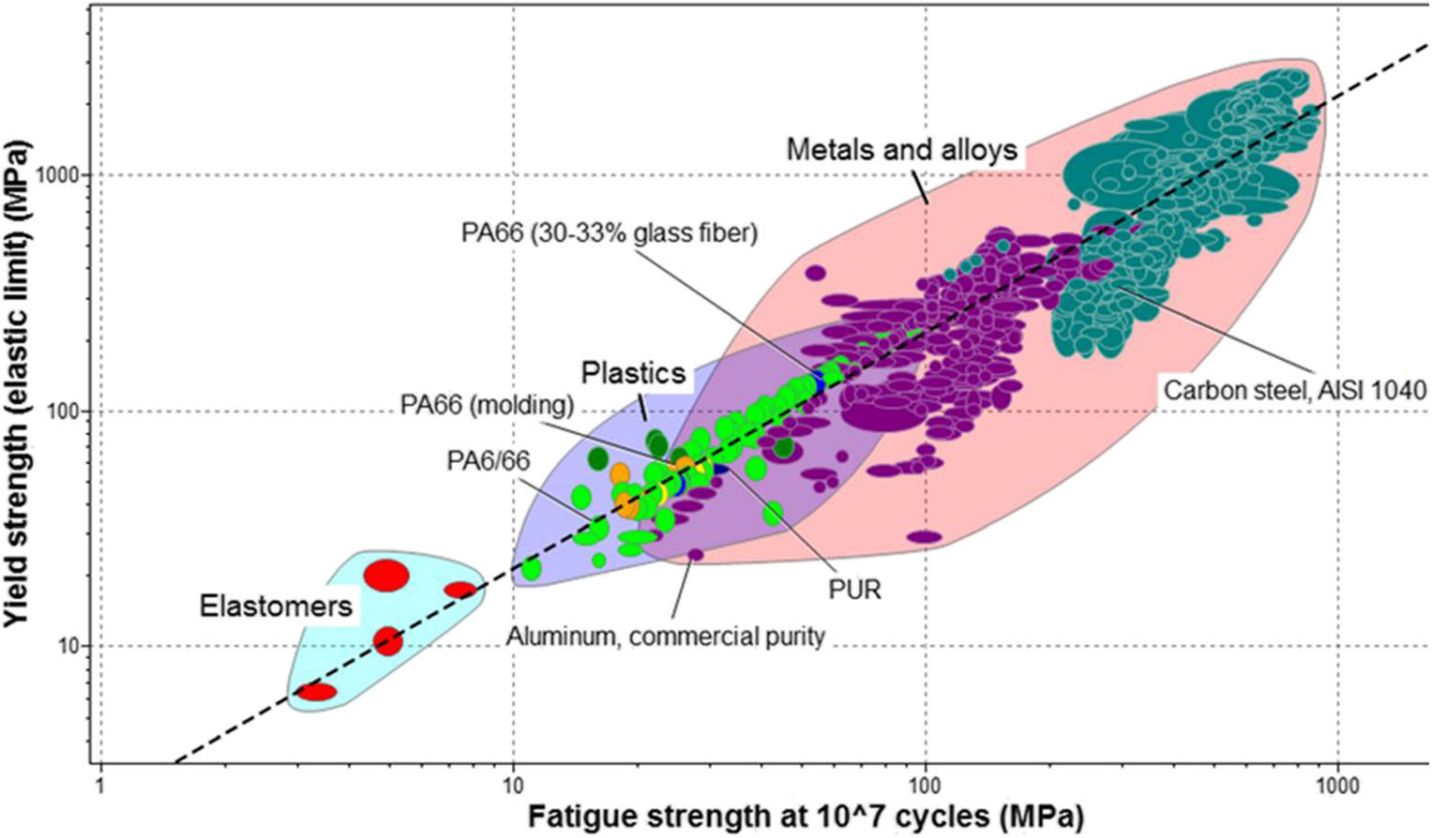
Comparison between yield strength and fatigue strength for different materials suitable for sheet metal tools

3D printed thermoplastics that could be used for sheet metal forming operations are summarized in Fig. 1, where two of the most important mechanical parameters (yield strength and fatigue strength) are shown, in comparison to metal materials and elastomers.

### Polymeric EAM rapid tools for injection molding

The demand for shorter development spans and reduced product lifecycle has augmented the interest in utilizing the rapid tooling techniques in injection molding. Different techniques have been employed for the rapid manufacturing of injection molds like selective laser sintering (SLS), direct metal laser sintering (DMLS), selective laser melting, fused deposition modeling, and polyjet 3D printing. However, rapid tooling techniques for injection molding also possess certain limitations in terms of mold material, accuracy, surface finish, and mold life [28].

Most of the commercial conventional FFF devices are used with ABS or PLA thermoplastic materials delivered as a filament from spools. Other material options include polycarbonate, polyamide, high-impact polystyrene, polyetherimide, polyoxymethylene, polyphenylsulfone, and some others. Recent works have further expanded the printable materials to include other polymer blends as well as ultra-high molecular weight polyethylene [29]. Numerous efforts have been made by the researchers to develop novel materials, composites for FFF, and by advancing the currently available materials by altering their properties and/or composition by addition of other materials. Work has been done by Nikzad et al. to produce filament of composite materials by proper formulation and mixture of constituent material [30].

In the field of rapid tooling for injection molding, FFF has also been used to produce ABS molds employed for wax injection molding at pressures and temperatures of 1.38 MPa and 66 °C respectively [31]. Another interesting example of rapid tooling for injection molding inserts through FFF is reported by Masood et al. in [32]. The authors realized inserts with iron particles in a nylon type matrix. The feedstock filaments of this composite have been produced and used successfully in the unmodified FFF system. High-quality plastic parts have been injection molded using the inserts. The work represents a major development in reducing the cost and time in rapid tooling.

Dizon et al. compared two molds for injection molding of PLA: one made from UV-cured resin by SLA and the other made up of ABS using FFF. They concluded that the UV-curable resin can be used as a mold for injection molding with good dimensional accuracies and the FFF-printed molds can be employed in direct rapid tooling in injection molding but for a limited number of shots [33].

Another interesting study was conducted by Altaf et al. [34], who 3D printed ABS mold using Cube Pro 3D printer for powder injection molding (PIM) of a bio-medical hip implant. The copper powder was used for PIM with poly-ethylene as the binder. Decent results were obtained, albeit with some defects in the final part.

**Fig. 2:**
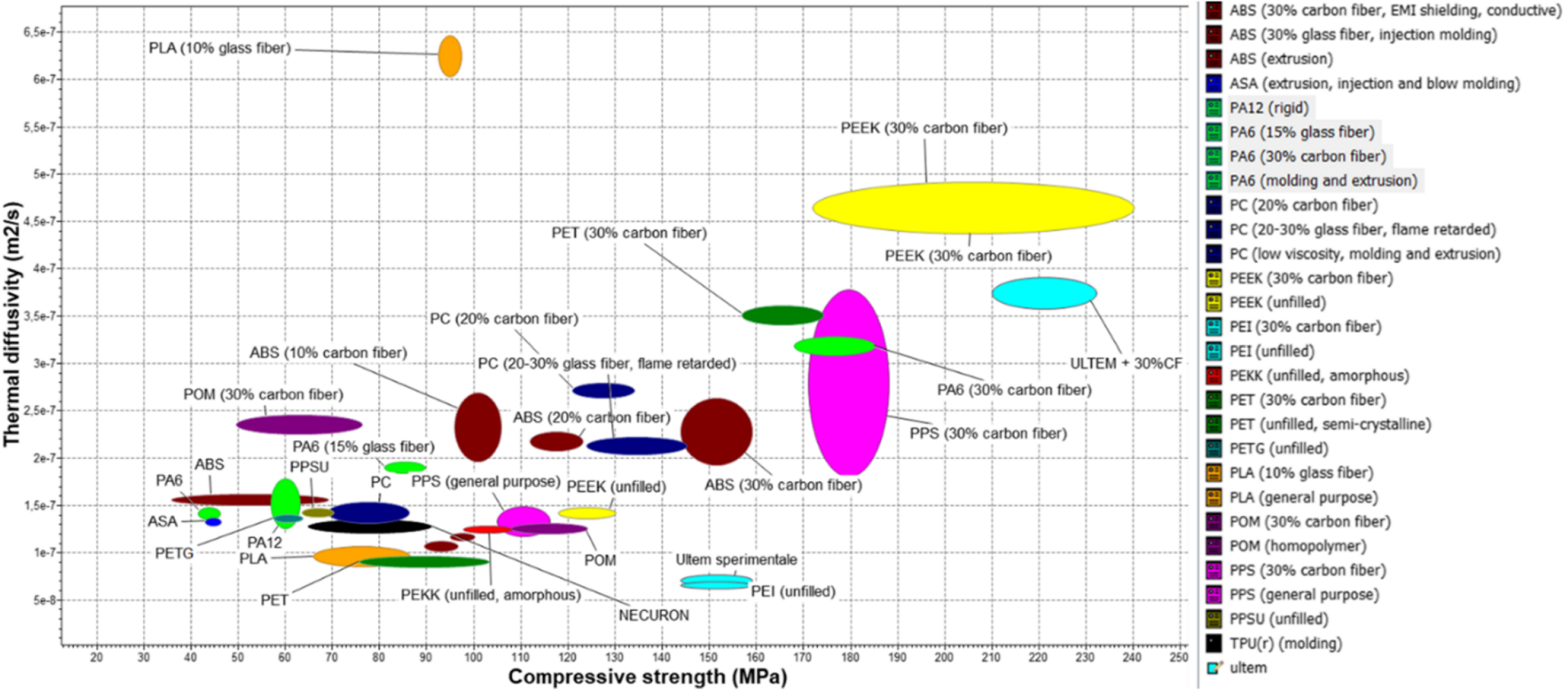
Comparison between thermal diffusivity and compressive strength some thermoplastic materials for injection molding molds

These promising studies motivate and highlight the necessity to take advantage of the EAM techniques by considering different mold materials for injection molding. The most important polymers that could be 3D printed by EAM and used as molds for injection molding applications are summarized in Fig. 2.

A recent study demonstrated [35] that 3D printed ULTEM (the polymer is PEI, polyetherimide) can sustain the mechanical and thermal loads involved in injection molding of glass fiber–reinforced polypropylene.

The above-stated thorough literature overview directs to the following conclusions:

- For sheet metal operations like shearing and blanking, as the tools must possess high hardness and be wear-resistant, so the most viable option is metallic tools as they provide the most optimal results and can be 3D printed by EAM using metallic feedstock.
- For sheet metal operations like bending and deep drawing, it is better to use a combination of metallic punches and polymeric dies as compared to metal-metal or polymeric-polymeric combination as confirmed by Nakamura et al. [21]. As the forces in the punch are much more concentrated, it must be made from metal to get optimum results. Consequently, EAM can be utilized to 3D print both the metallic punches and polymeric dies respectively.
- For injection molding, EAM provides a cost-efficient method to 3D print the tools by extruding a polymeric feedstock. Although PLA and ABS are the most widely used polymeric materials, EAM provides the flexibility to use any polymeric material thus expanding the prospect of polymeric tooling.

**Fig. 3:**
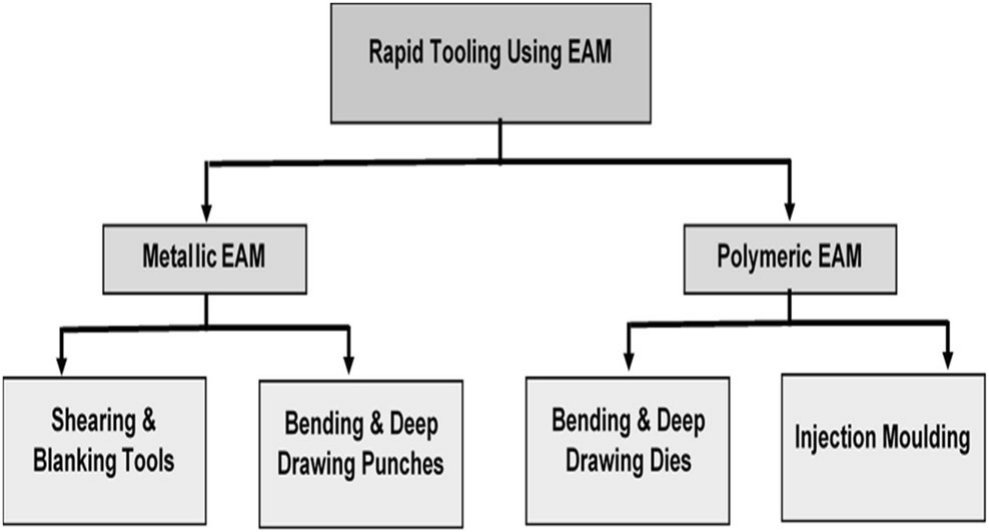
Hierarchy of EAM tooling applications in sheet metal and injection molding

The hierarchy of EAM tooling applications in sheet metal and injection molding applications is shown in Fig. 3.

## EAM process for metal tools

**Fig. 4:**
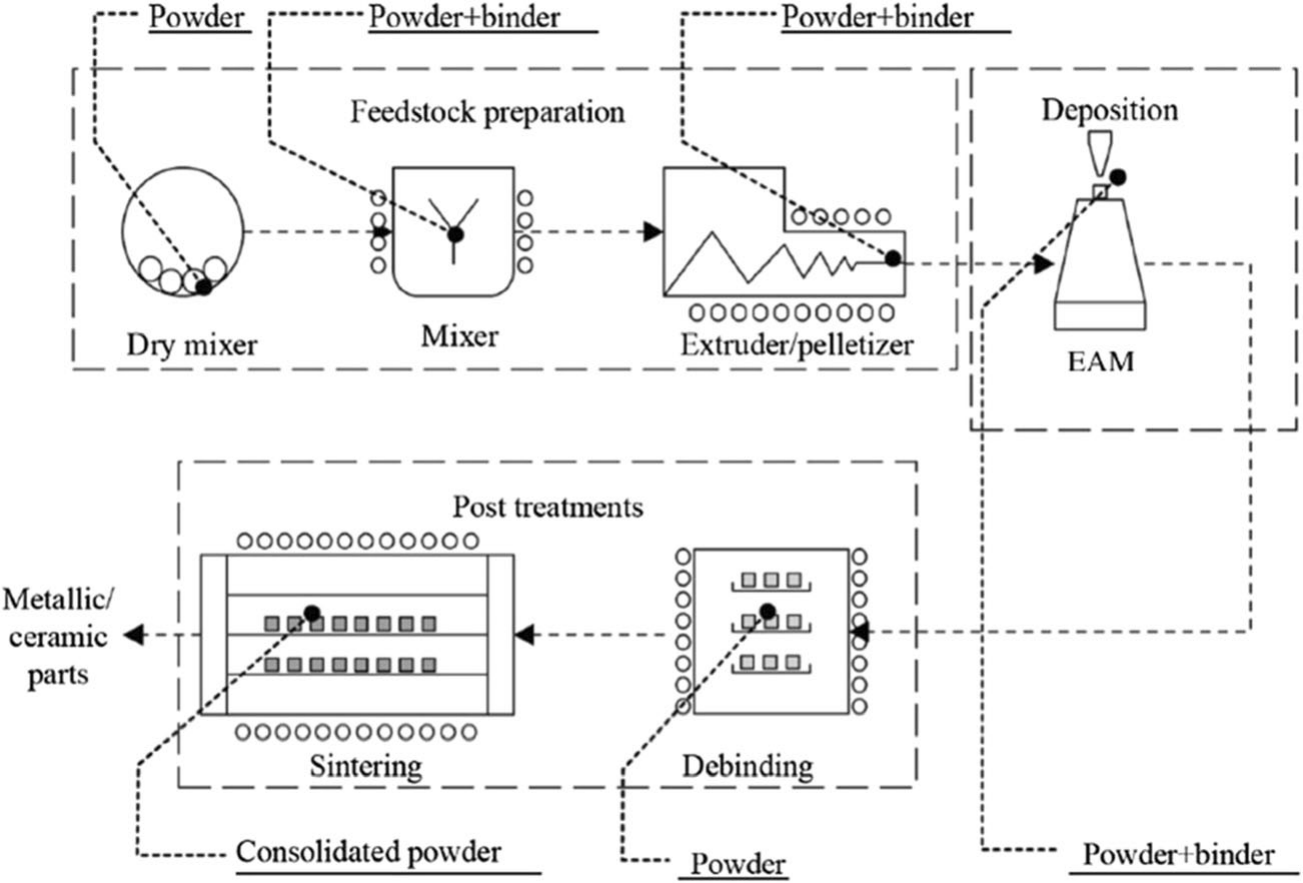
The EAM production cycle for metallic/ceramic test samples

Tests have been performed for producing sample parts, meant as rapid tools, made of HSS (high-speed steel) tool steel. The overall production process followed for the test samples is described as follows, as a sequence of steps, and illustrated in Fig. 4. In a variant of the cycle, an intermediate milling operation at the green state (i.e., after 3D printing) can be added. To shorten the cycle, the commercial feedstock is becoming more and more available, so that the mixing phases can be avoided.

### Feedstock preparation

**Fig. 5:**
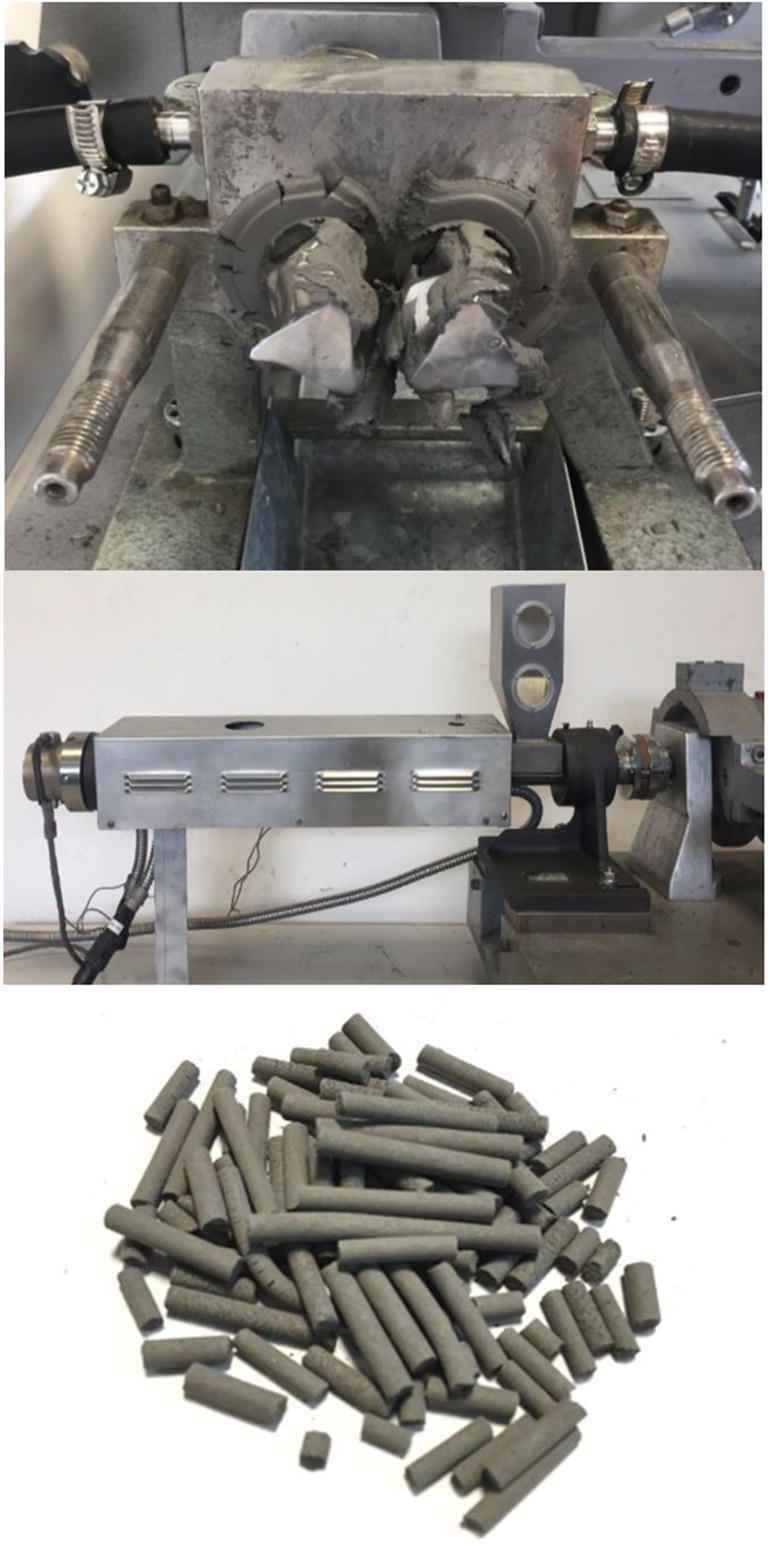
Brabender mixer (top), pelletizer (middle), and pelletized feedstock (bottom)

Steel powder and binder (typically 60% by volume) are first mixed to constitute the feedstock which is the starting material for this process. The best compounding result is reached with an extruder (Fig. 5) or a shear roll compactor. The most important properties of the feedstock are its homogeneity and rheological behavior. Only with optimal homogeneity, it is possible to manufacture faultless parts. The final part quality depends greatly on the rheological properties of the feedstock, the granulometry of the powder, and the viscosity at the extrusion temperature [36].

The used solid steel powder is supplied by Sandvik Osprey UK; the material is a T15 tool steel. The sieve analysis for the particle size of the material shows that all grains are less than 32 μm, with the 90th percentile d_90_<16 μm. The binder was supplied by eMBe; it was mixed at a weight fraction of 7.2% with the steel powder. It can be easily removed by the debinding cycle comprising of a water debinding process followed by a thermal debinding process.

Mixing has been performed with the Brabender machine (Fig. 5, top) with two counter-rotating screws, tightly placed in a mixing chamber. Powder and binder are mixed for 30 min with 100 rpm at 145 °C and then cooled down until 70 °C (for about 45 min) with 50 rpm. Then, the feedstock is extruded (Fig. 5 middle) as wire of 2.5 mm in diameter. Finally, the extruded wire is pelletized (Fig. 5 bottom).

### 3D printing

**Fig. 6:**
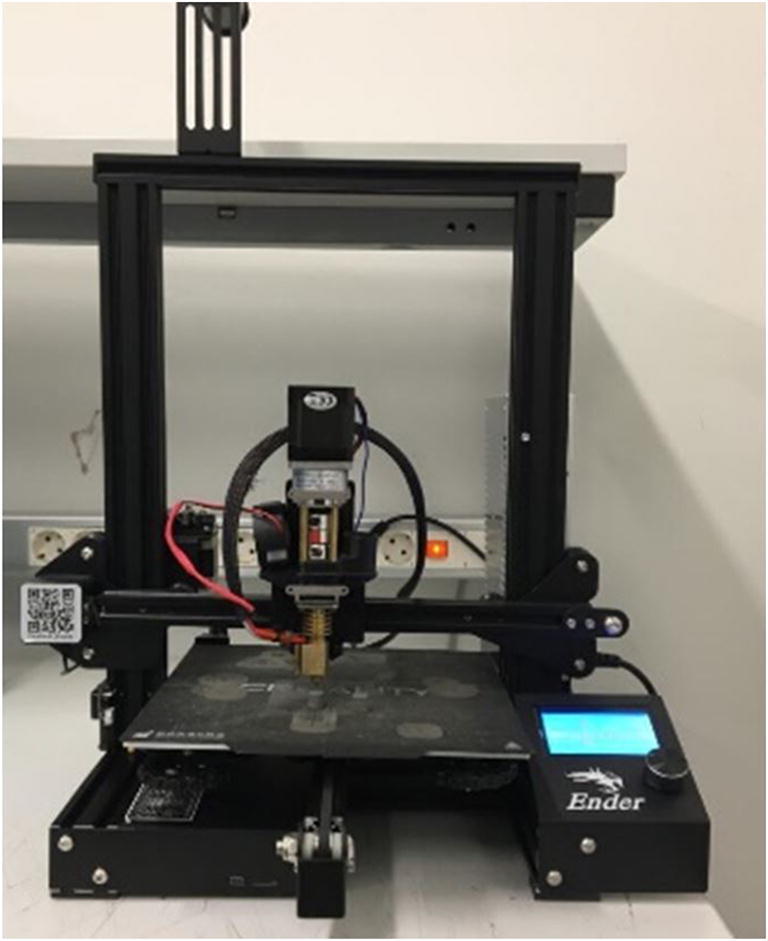
3D printer equipped with a pellet extruder

The feedstock has been 3D printed with a consumer type 3D printer (Ender-3 by Creality), equipped with a pellet extruder in place of the usual filament extruder (Fig. 6). First, a screening experimental plan was conducted to assess the preferable printing conditions.

**Fig. 7:**
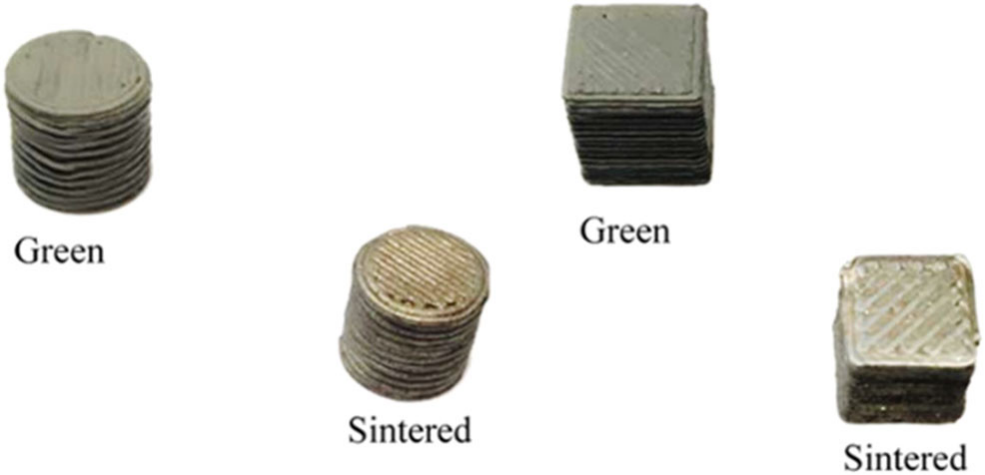
Examples of green and sintered specimens form the screening experimental plan. The side of the sintered cube is around 6 mm

In the screening plan, a total of 64 cubic or cylindrical samples (Fig. 7) were produced, by changing the following process parameters: nozzle tip diameter Dn, extruder temperature Te, extruder velocity Ve, layer height *h*, and shape orientation (cube and cylinder), while the infill density (100%), dimensions, and bed temperature (*T*_bed_) were kept constant. The used and the preferred printing parameters are given in Table 1. The dimension of the samples was taken and compared to the nominal dimensions. The resulting best combination of parameters that gives the minimum dimensional error is highlighted in Table 1 in bold font.

**Table 1:** Screening experimental plan. Highlighted in bold are the values that yield the best dimensional quality

Parameter	Symbol	Units	Values
Nozzle tip diameter	Dn	Mm	0.4, 0.6, 0.8
Extruder temperature	Te	°C	180, 200, 220
Layer height	h	Mm	0.3, 0.4
Shape			Cubic, cylindrical
Extruder velocity	Ve	mm/s	50, 60

### Debinding and sintering

T15 tool steel metallic parts, after 3D printing, require some post-processing stages: debinding process with two steps and sintering process with one step. The debinding process needs a solvent bath in still water for 96 h at 50 °C. The specimens must be fully submerged in the bath, then dried in an air convection furnace at 70 °C for 1 h, and finally cooled down at ambient temperature with desiccant. Then, thermal debinding at 280 °C (heating rate 10 °C/h) and sintering at 1280 °C (heating rate 120 °C/h) occurs in air atmosphere and argon flow, respectively, for a total of 45 more hours. In Fig. 7, the green samples (after 3D printing) and sintered samples (after debinding and sintering) for both cylindrical and rectangular samples are shown. Dimensional shrinkage can be clearly observed in Fig. 7 (measured linear shrinkage amounts to around 14%).

### Results of EAM process for metal tools

First of all, some relevant results of the screening plan will be presented.

#### Hardness

**Fig. 8:**
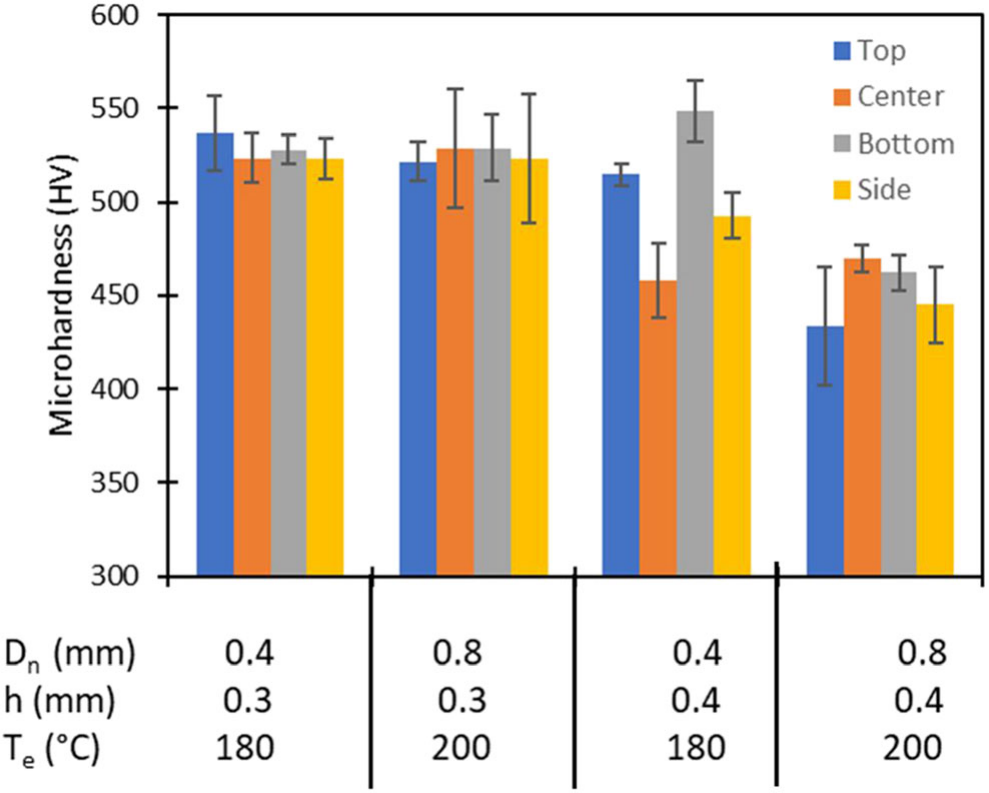
Hardness results of the screening plan: results of samples obtained under 4 different printing conditions

For metal tools, hardness is one of the most important properties. Cubic and cylindrical samples have been measured with 3 repeated measurements at each side of the samples. The results are shown in Fig. 8. Vickers hardness changes from a minimum of about 400 HV to a maximum of about 550 HV. These hardness values are totally in agreement with the typical hardness of sintered T15 steel.

#### Microstructure

**Fig. 9:**
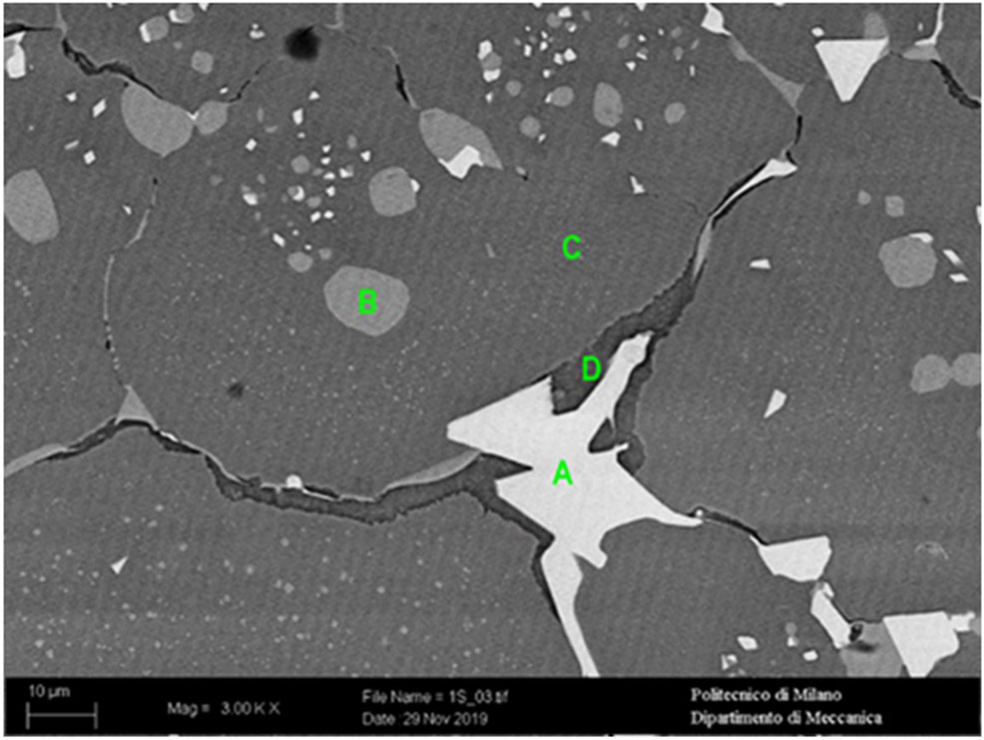
The microstructure of the best part

Scanning electron microscope (SEM) was used for microstructural characterization. A magnification of ×3000 was utilized for observing the microstructure of the best sample according to the screening test. The microstructure of the best part according to the screening test is not homogenous. There are some brighter regions, which do not possess uniform mechanical properties. There are four different regions, labeled as A, B, C, and D, in Fig. 9. Region A is the brightest and contains 64.55% of tungsten; region C is a matrix rich in iron, similar to standard composition; region B is richer with carbon (11%) whereas region D is richer with chromium (7%).

#### Sintered density

The theoretical density of T15 is 8.19 g/cm^3^ and the theoretical density of the green mixture is 7.67 g/cm^3^. The actual final density after sintering depends on the residual porosity inside the samples. The average measured density of the samples was 7.24 ± 0.21 g/cm^3^ (measured with the gravimetric buoyancy method in ethanol). It is low as compared to the theoretical density resulting in a relative density of 88% indicating the presence of porosity in the samples.

#### Surface roughness

**Fig. 10:**
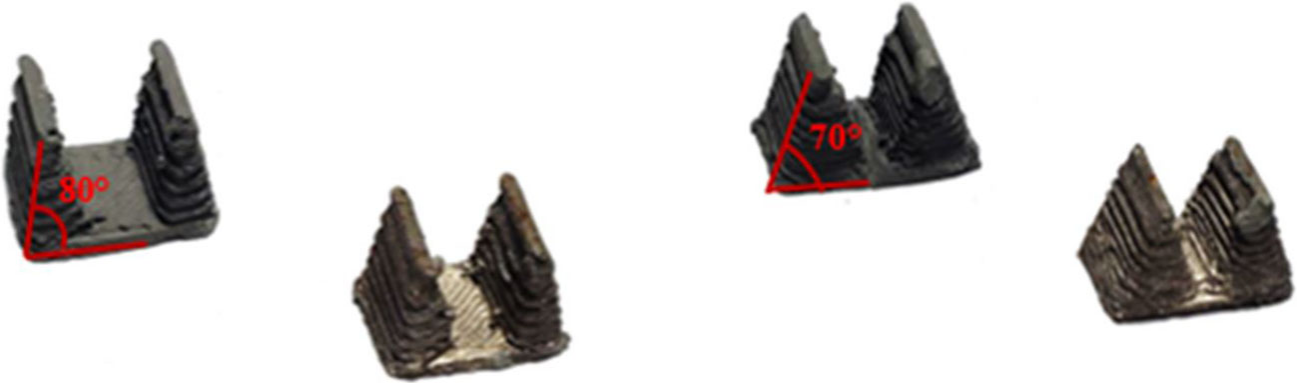
Hardness results of the screening plan

**Fig. 11:**
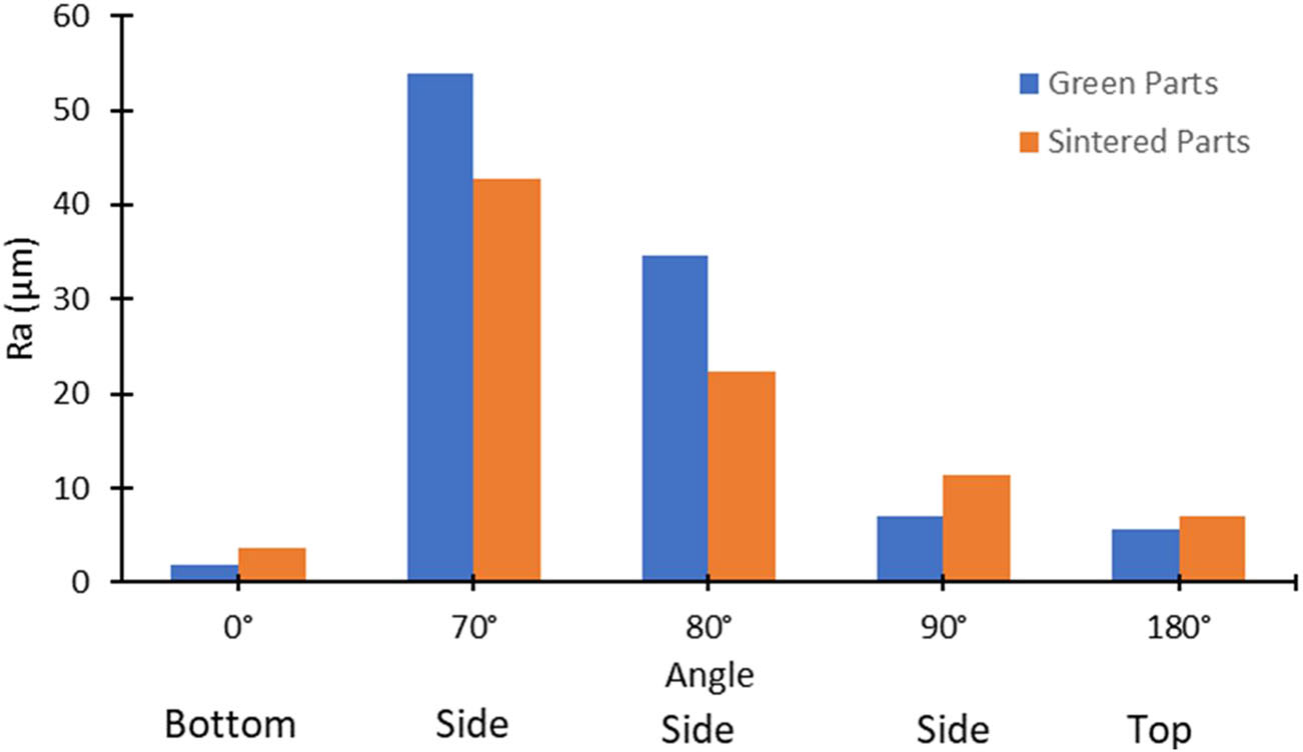
Hardness results of the screening plan

**Fig. 12:**
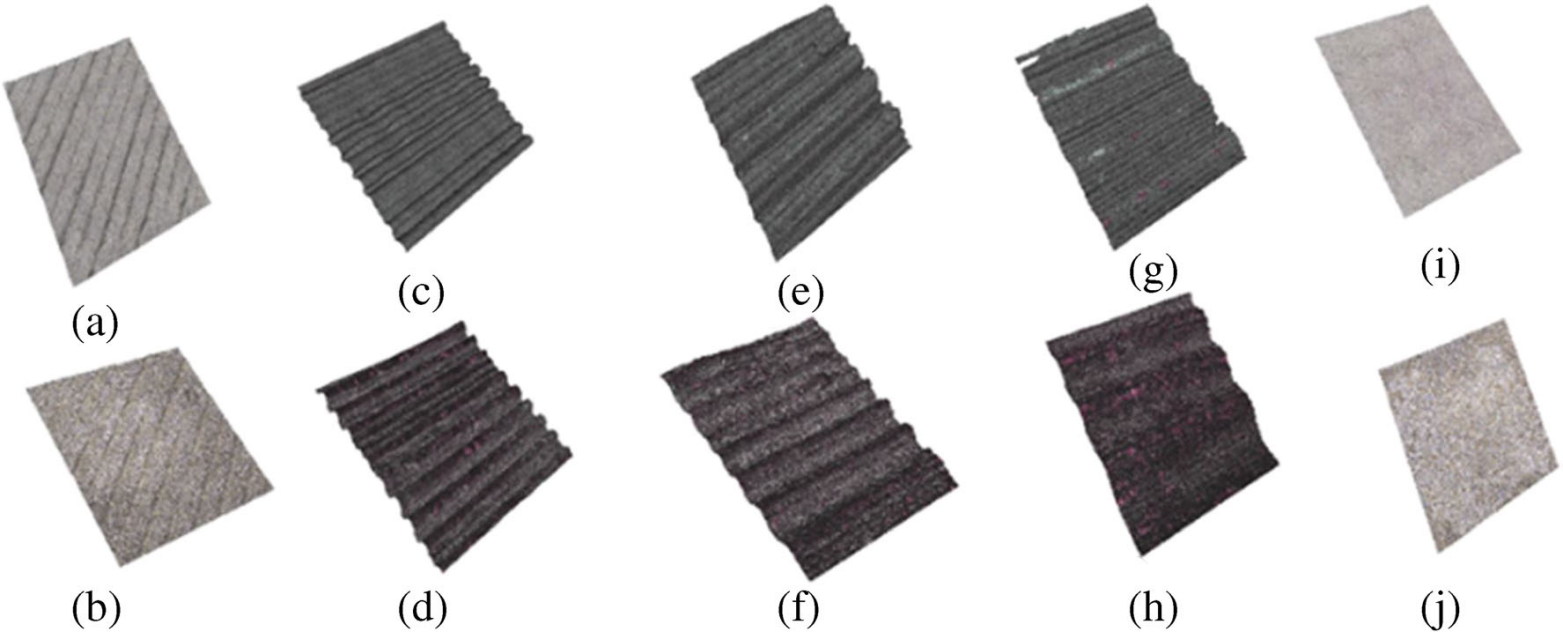
3D surface profiles **a** 180° green, **b** 180° sintered, **c** 90° green, **d** 90° sintered, **e** 80° green, **f** 80° sintered, **g** 70° green, **h** 70° sintered, **i** 0° green, and **j** 0° sintered. The scanned surface is a square with about 3-mm side

The surface roughness of samples has been measured with a Bruker Alicona InfiniteFocus Microscope over a 4-mm sample length. The arithmetic mean roughness Ra has been measured, along with a 3D reconstruction of the surfaces, in both the green and sintered states. Additional samples have been printed and sintered, with surfaces inclined at 70° and 80° (Fig. 10). Vertical and horizontal surfaces, as expected, have a better roughness. The staircase effect causes worse values for 70° and 80° surfaces (Fig. 11). The original roughness of green samples improves after sintering (Fig. 12).

### Examples of 3D printed metal tools

**Fig. 13:**
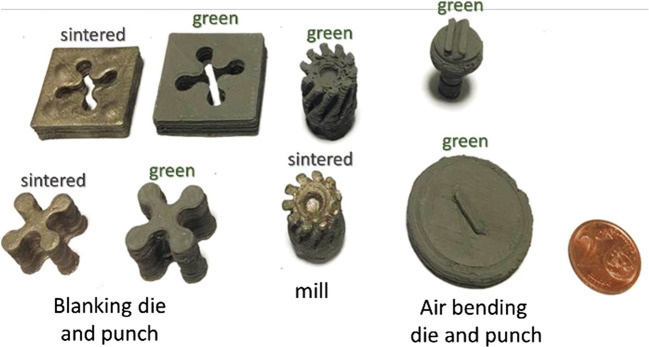
Green and sintered bending, milling, and blanking tools

Different materials for 3D printing of tools can be used depending on the application. For punching and other severe operations, metal powders can be used to 3D print metallic rapid tools using EAM whereas for less severe operations like bending and injection molding, high strength plastics can be used to 3D print rapid tools using EAM, thus depicting the versatility of the EAM techniques in the rapid tooling domain. By taking advantage of the best printing conditions determined at the previous steps, tests have been conducted to show the feasibility of producing prototypes of small, special tools for different tooling applications as shown in Fig. 13. The research demonstrates that it is feasible to produce metal tools in a cost-effective with very simple and low-cost 3D printing equipment. However, the surface quality and the dimensional precision of parts are not sufficient for precision application such as blanking. The surface quality and dimensional precision are also not sufficient for producing sharp and effective cutting tools. Finally, the dimensional precision and surface quality are barely sufficient to produce air bending or forming tools. In the case of blanking and bending tools, where the geometries do not have undercuts, the process can very easily and rapidly be modified to add a milling operation at the green state [32] to provide the desired tolerances. However, not only the surface and geometrical quality are a problem, but also the fatigue life of the tools might be reduced because of the largely obtained porosity. This means that the proposed tools will be able to work only for low batch productions.

### Remarks for EAM of metal tools

The following remarks can be drawn from the abovementioned attempt for 3D printing of metallic tools using EAM:

i) It is possible to 3D print different metal tools for different applications using extrusion-based additive manufacturing (EAM). EAM provides a cost-efficient setup for the manufacturing of metal tools and these tools can be utilized for carrying out sheet metal operations.
ii) Although EAM enables the 3D printing of metal tools, the size of tooling must be kept small due to the underlying limitations of sintering capabilities. Large samples cannot be effectively sintered which limits the capability of EAM to small-sized tools only.
iii) The surface quality and precision of the metal tools printed by EAM is inferior to metal tools manufactured by conventional manufacturing processes. However, this issue can be taken care of by machining the metallic tools at a green state before the debinding and sintering stages as confirmed in this study [37].

## EAM of polymeric tools for injection molding

An attempt [35] was made to 3D print polymeric tools using one of the EAM techniques namely fused deposition modeling (FFF). As already been discussed, FFF is the most utilized EAM technique for 3D printing of polymers and a number of commercial feedstocks compatible with the FFF process are available. But, as per the literature, the potential and suitability of very few of these available feedstocks have been tested yet for the 3D printing of polymeric molds. Among the available polymeric materials, like PEEK, PEKK, PPSU, PPS, and PEI, polyethylenimine (PEI) was chosen as the material for 3D printing inserts for injecting polyoxymethylene (POM). The material selection was based on tool cost minimization considering thermo-mechanical constraints.

Before actual testing, a 2D finite element model was used to simulate the thermo-mechanical response of the PEI insert under cyclic injections. It was concluded, as a result of simulations, that the material selected is thermally and mechanically stable for the application but only for a limited number of runs or prototypal applications due to much lower stiffness and thermal diffusivity with respect to metallic tools.

**Fig. 14:**
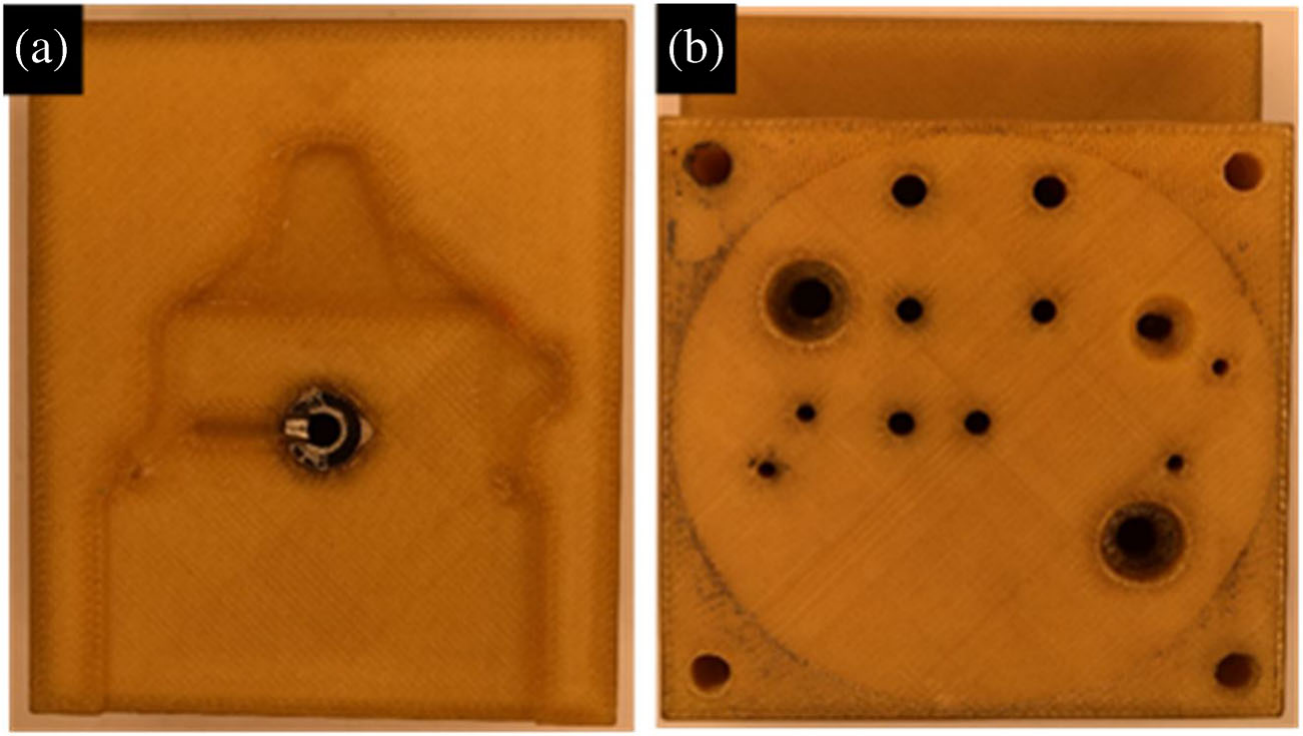
**a** Fixed inserts and **b** moveable inserts

**Fig. 15:**
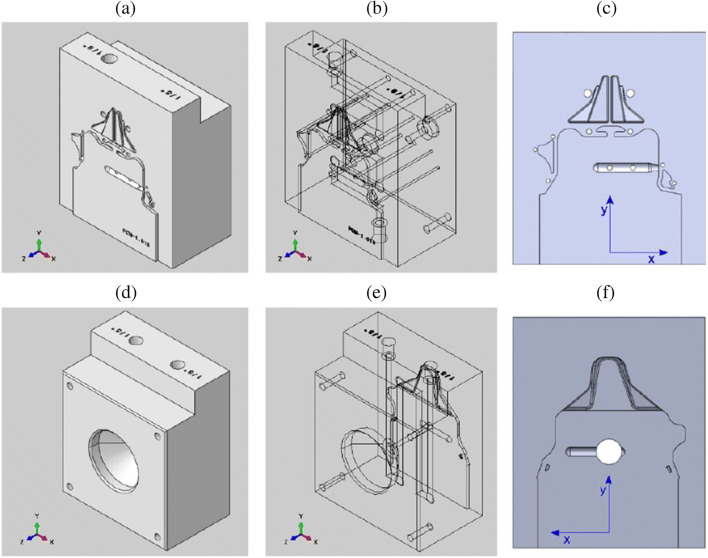
**a** Solid view of the movable insert. **b** Internal features of the movable insert. **c** Front view of the movable insert. **d** Solid view of the fixed insert. **e** Internal features of the fixed insert. **f** Front view of the fixed insert

In order to prove the real-life applicability of the tooling made of PEI, two inserts (movable and fixed molds) were 3D printed using FFF as shown in Fig. 14a and b for fixed ad moveable inserts respectively. These 3D printed inserts were identical to conventional metallic molds and contained all the usual features: the sprue, the runner, the gate, the cavity, guiding holes, cooling channels, holes for ejector pins, and a conical hole to mount the metallic sprue bushing shown in Fig. 15. The material was an ULTEM 1010 filament by Sabic.

**Fig. 16:**
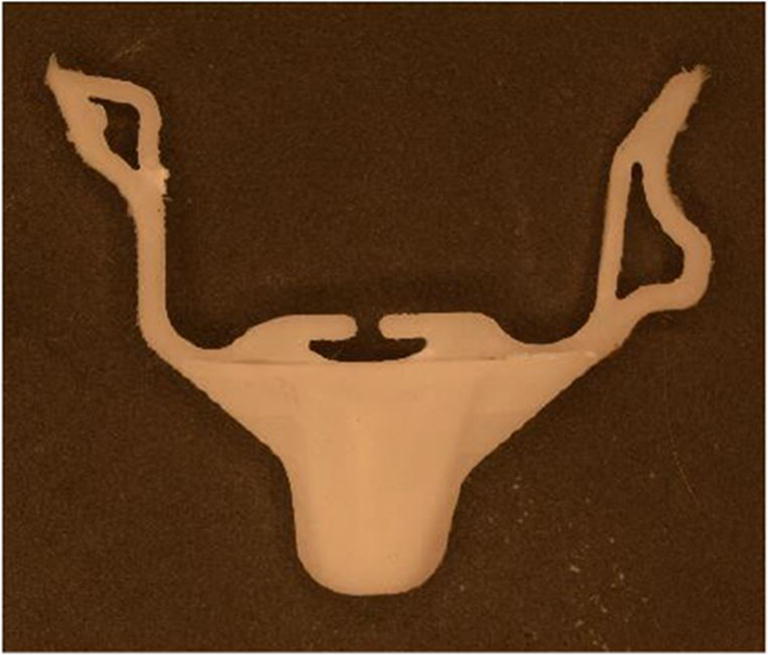
Molded part

These inserts were put under real-life testing conditions by mounting in a small injection molding unit (Babyplast) used for prototype and testing productions, and 20 POM parts were produced at 25 MPa packing pressure and 225 °C injection temperature, with a cycle time of 13 s. The inserts did not show any particular damages after these molding cycles. However, some other problems were observed like difficulty in de-molding the POM parts and water permeation through the inserts when the cooling system was active. The water permeation can be attributed to the inherited porosity of the 3D printed tools but this problem can be tackled by applying a thin and compact coating on the surfaces of the cooling channels, avoiding water permeation. The molded POM parts had a rough surface because the surface of the tools was not completely smooth which is a consequence of the FFF printing process (Fig. 16).

## EAM of polymeric dies for sheet metal

A study regarding the 3D printing of polymeric tools but for sheet metal applications was also carried out. The operation of V-die air bending of metallic (aluminum 5754 and mild steel AISI 1045) sheets was selected for this study and the material selected for the die manufacturing was polylactic acid (PLA, by Sharebot). Three sheet thicknesses 0.6 mm, 1.0 mm, and 2.00 mm were used for each sheet material. The study relied upon the compliance between a developed 2D model and the corresponding experimental setup. Different parameters (printing, geometrical and operational) were selected and the mechanical behavior of plastic tools was analyzed by several simulations to find the most optimum parameters providing the desired results for 3D printing of PLA dies for V-bending of metallic sheets.

In order to obtain the optimal dimensions of PLA based V-die, four different opening sizes were considered to investigate the effect of different sheet materials and their thicknesses on the stiffness of the die. Furthermore, five different raster orientations 0–90, 45–45, 45–0–45–90, 45–0–45, and 45–90–45 were considered for printing V-bending dies. These different die opening sizes (*W*), shoulder height (*h*) of the die, and the raster orientations are given in Table 2.

**Table 2:** Different combinations for die

Factors	Levels				
Die opening size (mm)	6	8	14	16	
Shoulder height (mm)	3	4.19	7.77	8.96	
Raster orientation	0– 90	45–45	45–0–45–90	45–0–45	45–90–45

**Fig. 17:**
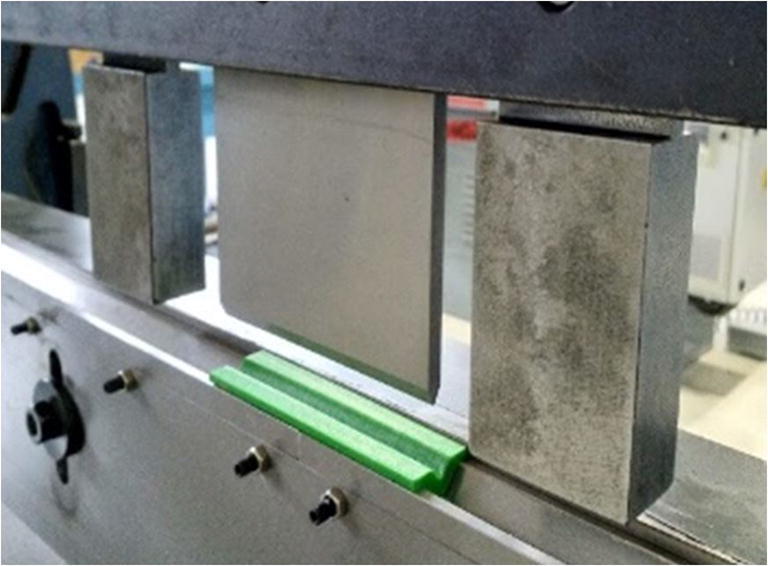
Experimental setup for V-bending test: the die insert is 80 mm long

**Fig. 18:**
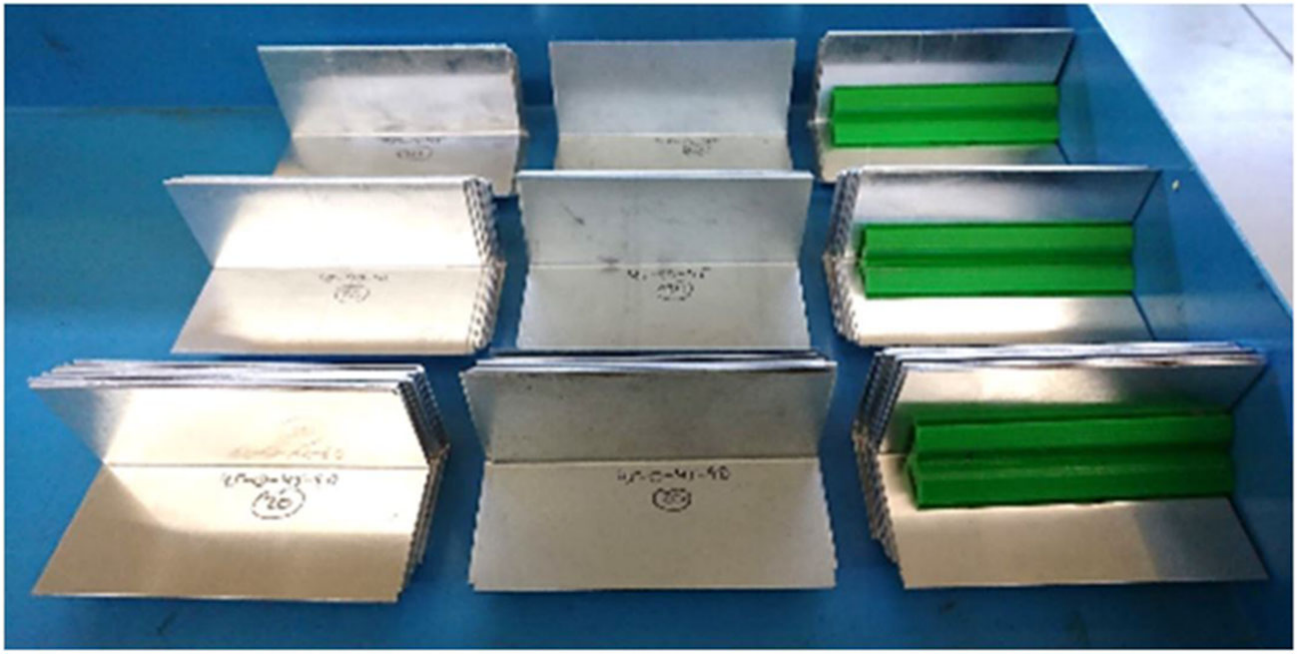
Sets of bent sheets

As a result of a number of simulations using the abovementioned combinations, it was established that the best strength was obtained using a raster orientation of 45–0–45 and a die opening size between 8 and 12 mm. Having established these parameters, a V-bending die made of PLA was 3D printed using FFF and put under real-life testing conditions for V-bending of aluminum and mild steel sheets having thicknesses of 0.6 mm, 1 mm, and 2 mm together with a steel punch. It was observed that the 3D printed plastic die displayed a similar spring back behavior in comparison with the traditional tooling. It was not possible to bend the 2-mm sheets using the PLA bending tools for both materials and the tooling is suitable only for short-run production. The experimental setup for V-bending tests and some bent sheets are shown in Figs. 17 and 18 respectively [38].

The results have shown that the PLA tools were able to sustain more than 100 repeated bends with no apparent deterioration nor macroscopic change in the obtained bend angle. The process variability of the obtained bent angle was within ± 0.6°, i.e., comparable to a conventional air bending process performed with metal inserts.

## Conclusions

This paper aimed at providing an insight into the potential and versatility of extrusion-based additive manufacturing (EAM) techniques for 3D printing metallic and polymeric rapid tools for sheet metal and injection molding applications. The paper highlighted the significant past works done by the researchers in the domain of rapid tooling using AM. Several production routes were proposed and discussed to produce rapid tools, by additive manufacturing, made of metal or plastics. Examples of 3D printing of both the metallic and polymeric tools were described. The materials used were tool steel for metallic tools and polyethylenimine (PEI) and polylactic acid (PLA) for plastic tools respectively. First, the possible production process of metal tools made of tool steel was presented, starting from the EAM (extrusion-based additive manufacturing) of a feedstock which is a mixture of metal powder and a polymeric binder. Then the results of the EAM process for metal tools were described. After that, the 3D printing process of PEI inserts for injection molding of POM was described. The results obtained in terms of final part quality and limitations of both the processes were discussed and finally, 3D printing of another plastic (PLA) tooling using FFF was described but for V-bending of metal sheets.

The paper has shown that EAM is a viable option for producing metal and polymeric tools for different tooling applications, although with limitations to fatigue life due to the inherent porosity of the obtained samples.

## Data Availability

The availability of data and materials is not applicable to this manuscript.
